# Kinetic Transition in Amyloid Assembly as a Screening Assay for Oligomer-Selective Dyes

**DOI:** 10.3390/biom9100539

**Published:** 2019-09-27

**Authors:** Jeremy Barton, D. Sebastian Arias, Chamani Niyangoda, Gustavo Borjas, Nathan Le, Saefallah Mohamed, Martin Muschol

**Affiliations:** Department of Physics, University of South Florida, Tampa, FL 33620, USA; jeremybarton@mail.usf.edu (J.B.); sebasdarias95@gmail.com (D.S.A.); chamani@mail.usf.edu (C.N.); gustavo.jesus.borjas@emory.edu (G.B.); nathanle@mail.usf.edu (N.L.); smohamed2@mail.usf.edu (S.M.)

**Keywords:** oligomer formation, amyloidosis, assembly pathway, dye fluorescence, thioflavin T

## Abstract

Assembly of amyloid fibrils and small globular oligomers is associated with a significant number of human disorders that include Alzheimer’s disease, senile systemic amyloidosis, and type II diabetes. Recent findings implicate small amyloid oligomers as the dominant aggregate species mediating the toxic effects in these disorders. However, validation of this hypothesis has been hampered by the dearth of experimental techniques to detect, quantify, and discriminate oligomeric intermediates from late-stage fibrils, in vitro and in vivo. We have shown that the onset of significant oligomer formation is associated with a transition in thioflavin T kinetics from sigmoidal to biphasic kinetics. Here we showed that this transition can be exploited for screening fluorophores for preferential responses to oligomer over fibril formation. This assay identified crystal violet as a strongly selective oligomer-indicator dye for lysozyme. Simultaneous recordings of amyloid kinetics with thioflavin T and crystal violet enabled us to separate the combined signals into their underlying oligomeric and fibrillar components. We provided further evidence that this screening assay could be extended to amyloid-β peptides under physiological conditions. Identification of oligomer-selective dyes not only holds the promise of biomedical applications but provides new approaches for unraveling the mechanisms underlying oligomer versus fibril formation in amyloid assembly.

## 1. Introduction

Deposits of amyloid protein fibrils with their characteristic cross-β sheet architecture are the dominant pathological feature associated with a wide variety of age-related human disorders [1,2,3,4,5,6,7]. Most prominent among these age-related amyloid diseases are Alzheimer’s disease and type-II diabetes [8,9,10,11]. Accumulating evidence has implicated transient populations of small globular oligomers (gOs), first discovered during in vitro growth of amyloid proteins, as perhaps the most dominant contributors to the clinical symptoms of amyloid diseases [12,13,14,15,16,17,18,19,20]. One of the major obstacles towards elucidating whether and how amyloid oligomer and fibril formation are linked and in ascertaining how oligomers affect the etiology of disease symptoms is the limited range of techniques for detecting and discriminating oligomers from concurrent amyloid fibrils. Current techniques for detecting and characterizing amyloid oligomers include atomic force microscopy (AFM) [21,22,23], photo-induced, cross-linking of unmodified proteins (PICUP) [24], mass spectrometry [25], optical spectroscopy [26,27] and, most widely used, immunostaining with conformation-dependent anti-oligomer antibodies [28,29]. However, with the exception of anti-oligomer antibodies, none of the aforementioned techniques lend themselves for oligomer detection ex vivo, let alone in vivo. In addition, they all suffer from poor temporal resolution, which is a serious obstacle for kinetics studies to reveal the role of oligomers in fibril formation. These limitations on detecting, quantifying, and monitoring oligomers, combined with the intrinsically transient character of amyloid oligomers, has hampered efforts at unravelling the basic mechanisms underlying oligomer formation, at elucidating their mechanisms of cell toxicity, or at correlating disease symptoms with the accumulations of oligomers in vivo. Yet, these are critical steps towards establishing the role of oligomers in disease etiology and for developing targeted therapeutic interventions.

Small molecules with a high selectivity for detecting amyloid oligomers over late-stage fibrils would be particularly useful. The benefits of small fluorophores, in particular, is highlighted by the commonly used amyloid indicator thioflavin T (ThT), with its high fluorescence enhancement in the presence of amyloid fibril [27,30,31]. It offers superior temporal resolution and sensitivity for monitoring fibril growth kinetics, which has been critical to the development of molecular models of amyloid fibril nucleation and growth [30,32,33]. Its membrane-permeant variant thioflavin S, in turn, is a commonly used histological amyloid stain [34,35]. ThT has also served as a template for the amyloid-binding moiety of the first positron emission tomography (PET) imaging probe Pittsburgh compound B [36]. However, ThT’s fluorescence response and binding affinity are strongly skewed towards late-stage fibrils over oligomers [27]. Hence, identification of novel oligomer-selective dyes (OSDs) would offer an important new modality for monitoring amyloid oligomer formation in vitro and for developing probes for oligomer detection in vivo [37]. Several such dyes have been tested and developed, often using fluorescence spectroscopy correlated with high-resolution imaging to establish oligomer selectivity [38,39,40]. However, so far they have found limited acceptance—partially due to the need for custom synthesis, the lack of commercial availability, or limited applicability. Ideally one would like a straightforward and reliable assay for screening a large set of dyes for select responses to amyloid oligomers over fibrils. Such efforts are hampered by the challenge of generating oligomer preparations not contaminated by fibril admixtures, and of stabilizing the intrinsically transient and metastable oligomers.

**Figure 1:**
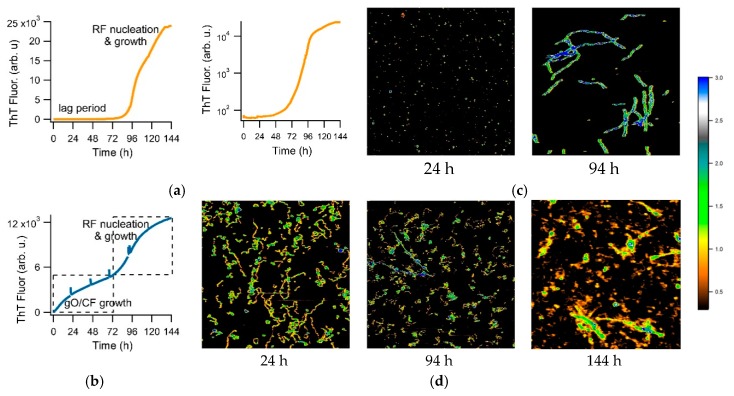
Sigmoidal versus biphasic amyloid kinetics and corresponding aggregate morphologies. Amyloid growth kinetics of hen egg-white lysozyme (hewL), recorded with thioflavin T (ThT), displaying either (**a**) sigmoidal (21 μM hewL) or (**b**) biphasic (350 μM hewL) growth kinetics (pH 2, 52 °C, 400 mM NaCl). Second panel in (**a**) is the same data on a semi-log scale to highlight the flat baseline. Spikes in the traces mark the times when aliquots were collected for imaging. Atomic force microscopy (AFM) images of the aggregate morphologies observed during (**c**) sigmoidal versus (**d**) biphasic growth of hewL, at the indicated time points. False color height scale: 3 nm.

Here, we investigate the use of a transition in amyloid kinetics induced by oligomer formation, which circumvents the need for isolating amyloid oligomers, to screen for oligomer-selective dyes (OSD). Specifically, we found that the formation of metastable globular oligomers is accompanied by the onset of biphasic growth kinetics [41]. We have previously shown that amyloid assembly of hen egg-white lysozyme (hewL) and of a dimeric Aβ40 construct (dimAβ), as recorded with the indicator dye thioflavin T (ThT), undergo a sharp transition from essentially oligomer-free sigmoidal to oligomer-dominated biphasic kinetics, upon crossing a (protein and salt concentration dependent) threshold [41,42]. Figure 1a shows sigmoidal kinetics recorded with ThT for hewL under the amyloid growth conditions used for subsequent experiments. An extended lag period, indicated by a complete lack of a ThT increase (notice semi-log scale) is followed by a rapidly accelerating upswing and plateau. AFM images document the absence of any discernible populations of prefibrillar nuclei, during the lag periods (Figure 1b, 24 h), with increasing numbers of progressively longer rigid fibrils emerging during the rapid upswing in ThT (Figure 1b, 94 h). Figure 1c displays an example of well-developed biphasic ThT kinetics emerging at higher hewL concentrations. Then, ThT fluorescence increases immediately and reaches an initial plateau, which is followed by a second upswing and plateau. This switch in assembly kinetics is associated with a dramatic change in observable aggregate morphologies. The initial phase of biphasic growth yields significant quantities of globular oligomers (gOs), which subsequently polymerize into curvilinear fibrils (CFs) (Figure 1D, 24 & 94 h). Rigid fibrils (RFs) only gradually emerge following the second upswing in ThT, with gO/CFs persisting long into the second plateau phase. (Figure 1D, 94 & 144 h).

We have provided evidence that this transition is due to a micelle-like concentration threshold for the gO/CFs formation which we called “critical oligomer concentration”. This matches with prior reports of a “critical micelle concentration” (or CMC) for oligomer formation in Aβ42 [43,44] and with the biphasic kinetics associated with the formation of long-lived oligomers in Aβ40 [41,42,45]. In addition, we confirmed that these gO/CFs were metastable. Intriguingly, they also retarded the kinetics of subsequent RF nucleation and growth, presumably by reducing secondary fibril nucleation rates and by buffering monomers. The gO/CF-induced retardation of RF formation is discernible by comparing the ThT amplitudes at the end of the biphasic (Figure 1a) versus sigmoidal (Figure 1c) growth. The ThT amplitude of the high concentration biphasic solution, at the end of the incubation period, reached only about 1/2 the ThT amplitude for the low-concentration sigmoidal solution. In addition, the RFs emerging during the biphasic growth were decorated by gO/CFs. We have argued that it is this interactions between RFs and gO/CFs that retards RF secondary nucleation [41]. For the purpose of the subsequent dye screening assay, though, we only relied on the fact that sigmoidal kinetics indicates essentially oligomer-free RF formation, while the initial phase of biphasic growth is dominated by gO/CF formation. To detect oligomer-specific dye responses, we identified growth conditions for which the oligomer-dominated phase of biphasic growth developed a plateau, separated in time from subsequent fibril nucleation and growth (see Figure 1b).

**Figure 2:**
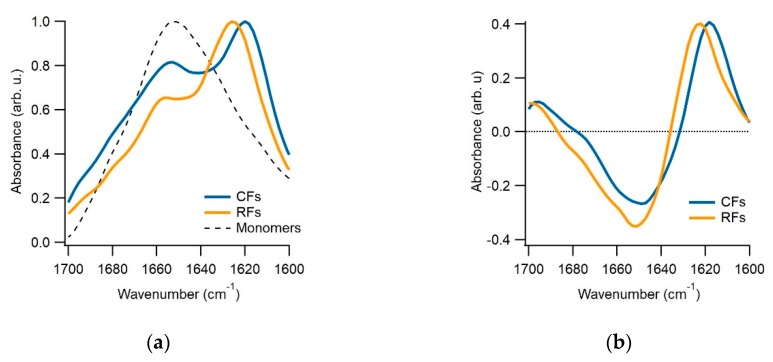
FTIR spectra of hewL monomers, curvilinear fibrils (CFs) and Rigid fibrils (RFs). (**a**) Peak-matched FTIR absorption spectra of the Amide-I band for hewL monomers and isolated CFs and RFs at pH 2. CFs and RFs show a prominent peak in the characteristic amyloid band between 1630 and 1610 cm^−1^. Yet their peak wavenumbers are slightly but reproducibly shifted with respect to each other. (**b**) The CF and RF differences spectra obtained after subtraction of the monomer spectrum in (**a**).

We have multiple pieces of evidence to indicate that the gO/CFs emerging during the biphasic growth of either hewL or dimAβ match the properties of toxic oligomers associated with amyloidoses. First and foremost, gO/CFs are early-stage metastable aggregates which become eventually superseded by the ‘traditional’ late-stage RFs [41,42]. Their metastability is highlighted by the initial plateau phase seen in the ThT traces (see Figure 1b) and their prolonged persistence, even during the late stages of RF growth. The gO/CFs also display the well-defined globular morphology of toxic oligomers which, in the case of hewL, are composed of about eight monomers [23]. These gOs subsequently polymerize to form CFs, with cross-sections identical to those of their octameric building blocks, and distinct from the monomeric cross-section of the RF filaments [23,46]. Again, CFs are structures frequently associated with toxic oligomeric preparations. Both CD and FTIR further indicate that these early-stage gO/CFs share the spectroscopic signatures of amyloids. In FTIR, gO/CFs display a prominent peak in the Amide I “amyloid band” between 1610 and 1630 cm^−1^ [47]. Yet the peak wavenumber is distinct from RFs and typically shows additional absorption around 1690 cm^−1^, which is considered indicative of antiparallel β-sheet structures [26,27]. FTIR spectra for hewL RF and gO/CFs used in our experiments are shown in Figure 2. They match our previous observations and replicate spectroscopic features of Aβ oligomers and their proposed 3D structure [26,27,48]. Similarly, the noticeable but muted ThT amplitudes associated with gO/CF formation, particularly when compared to equivalent concentrations of RFs, matches the weak ThT responses to gO/CFs for a wide range of oligomeric amyloid species [23,27,41,42,46]. Oligomers of hewL have also been shown to react with oligomer-specific antibodies and induce neurodegeneration, when injected into rat brains [49]. Hence, the gO/CFs emerging during the initial phase of biphasic growth share basic characteristics of toxic oligomers seen with various amyloid-forming proteins and peptides—even if the specific in vitro conditions used to generate them during the biphasic growth do not match those present in vivo. It is worth noting that the initial discovery of toxic oligomers, subsequent assays for their generation, the biophysical characterization, as well as the development of anti-oligomer antibodies, similarly, relied on in vitro growth of oligomers under various, often non-physiological growth conditions.

Here, we evaluated the responses of fluorescent dyes to RF-dominated sigmoidal versus gO/CF dominated biphasic growth, as an assay to screen for oligomer-selective dyes (OSDs). Specifically, we used sigmoidal versus biphasic growth conditions of hen egg–white lysozyme, during amyloid assembly at pH 2 (Figure 1), to investigate the oligomer-selectivity for a small set of fluorescent dyes. For the most promising candidate, we identified the triarylmethane dye crystal violet (CV), we further investigated whether its apparent oligomer selectivity instead arose from interference with fibril assembly. We next determined whether CV could be used for simultaneous recordings of amyloid kinetics with ThT and, therefore, for reconstructing the separated contributions of oligomers versus fibrils to the compound dye kinetics. To expand our approach, we established that the two Alzheimer’s disease peptides, Aβ40 and Aβ42, undergo a similar kinetic transition under physiological growth conditions. This indicates that the proposed screening approach, tested here for dye responses during amyloid formation of a non-human protein under non-physiological growth conditions, can be readily extended to screen for OSDs for the two main peptides implicated in Alzheimer’s disease.

## 2. Materials and Methods

### 2.1. Protein and Chemicals

Two times recrystallized, dialyzed, and lyophilized hen egg white lysozyme (hewL) was purchased from Worthington Biochemicals (Lakewood, NJ, USA) and Aβ40 was purchased from rPeptide (Watkinsville, GA, USA). Ultrapure grade Thioflavin T (ThT) was obtained from Anaspec (Freemont, CA, USA). Crystal violet (CV) and other chemicals were from Fisher Scientific (Pittsburgh, PA, USA) and were of reagent grade or better. All solutions were prepared using 18 MΩ water from a reverse osmosis unit (Barnstead E-pure, Dubuque, IA, USA).

### 2.2. Preparation of HewL, Aβ40, Aβ42, CV, and ThT Solutions

Lyophilized hewL was dissolved at twice its final concentration in a 25 mM KH_2_PO_4_ pH 2 buffer, with any residual aggregates dissolved by incubating the solution for a few minutes at 42 °C. [50] Samples were typically filtered through 220 nm PVDF (Fisherbrand, Fisher Scientific, Pittsburgh, PA, USA) and 50-nm PES (Tisch Scientific, North Bend, OH, USA) pore size syringe filters. The resulting solutions were highly monodisperse, as assessed with both dynamic light scattering and SDS–PAGE [50]. A series of hewL solutions at different concentrations was generated by diluting this hewL/buffer stock with buffer, to twice its final hewL concentration and then mixing it 1:1, with a NaCl/25 mM KH_2_PO_4_ pH 2 stock solution at twice the final salt concentrations. Actual lysozyme concentrations were determined from UV absorption measurements at 280 nm (ε_280_ = 2.64 mL mg^−1^ cm^−1^). Solutions for a series of hewL (or NaCl) concentrations spanning the transition from sigmoidal to biphasic behavior were prepared, and triplicates of each solutions were placed in 96-well glass bottom plates and were sealed. Lyophilized Aβ40 or Aβ42 peptide was dissolved in 100 mM NaOH and injected into a Superdex 75 10/300 GL column on an fast protein liquid chromatography (FPLC) system (Äkta Pure, GE, USA), using a 35 mM Na_2_HPO_4_ running buffer at pH 11. The monomer fraction was collected and kept on ice. The resulting Aβ monomer concentrations were measured using optical absorption at 280 nm with ε_280_= (1470 ± 20) M^−1^ cm^−1^ [51,52]. This Aβ stock was diluted into ice-cold 35 mM Na_2_HPO_4_ at pH 11 at the highest Aβ concentration and the pH was adjusted to pH 7.4, by addition of 1.5% (by vol.) of 1M NaH_2_PO_4_.

CV and ThT stock solutions were prepared at a concentration of 1 mM dye in distilled water and passed through 220 nm syringe filters. Actual dye stock concentrations were determined from absorption using ε_590_ = 87,000 M^−1^ cm^−1^ for CV and ε_412_ = 28,800 M^−1^ cm^−1^ for ThT, respectively. ThT and CV were added to protein solutions, either separately or together, as indicated. Unless indicated otherwise, ThT concentrations were 15 μM and CV concentrations were 5 μM.

### 2.3. Measuring Amyloid Growth Kinetics with Thioflavin T and Crystal Violet

Using a Fluostar Omega plate reader (BMG Labtech, Offenburg, Germany), hewL or Aβ 40 amyloid growth kinetics were measured during incubation at 52 °C (hewL) or 27 °C (Aβ 40), respectively. CV fluorescence was excited using built-in bandpass filters centered at 544 or 584 nm, respectively, with emission measured with a 620 nm bandpass filter. ThT fluorescence was excited with a 448 nm bandpass filter and emission was collected using a 482 nm bandpass filter.

### 2.4. Atomic Force Microscopy

Amyloid aggregates were imaged in air with a MFP-3D atomic-force microscope (Asylum Research, Santa Barbara, CA, USA) using NSC36/NoAl (Mikromasch, San Jose, CA, USA) or PFP-FMR-50 (Nanosensor, Neuchatel, Switzerland) silicon tips with a nominal tip radii of 10 nm and 7 nm, respectively. The cantilever had a typical spring constant and resonance frequency of 2 nN/nm and 70 kHz, respectively. It was driven at 60–70 kHz in alternating current mode and at a scan rate of 0.5 Hz, acquiring images at 512 × 512 pixel resolution. Raw image data were corrected for image bow and slope. For imaging, 75 μL of the sample solution was typically diluted 20- to 100-fold into the same salt/buffer solution used during growth, deposited onto freshly cleaved mica for 3–5 min, rinsed with deionized water, and dried with dry nitrogen. Amplitude, phase, and height images were collected for the same sample area. False-color heights were subsequently superimposed over either amplitude or phase images, offline.

### 2.5. Transmission Electron Microscopy

Amyloid samples were diluted into distilled water and 5 µL of the diluted sample was placed on Formvar/carbon-film-coated, 200 mesh copper grids and was allowed to air dry. A 5 µL aliquot of distilled water was added to the grid and blotted 3 times, to dissolve salt crystals. The grids were negatively stained with 5 µL of 8% (*w*/*v*) uranyl acetate, for 1 min, and were then blotted. Excess uranyl acetate was removed by repeated washing with distilled water and the grids were left to air dry. The grids were imaged using an FEI Morgani transmission electron microscope at 60 kV with an Olympus MegaView III camera (Center Valley, PA, USA).

### 2.6. Fourier-Transform Infrared Spectroscopy (FTIR)

Aggregates were prepared by growing them in a heat block at 52 °C. To remove monomer contributions to FTIR spectra, aggregate solutions were centrifuged in 2 mL centrifuge tubes at 15,000× *g* overnight. The resulting aggregate pellet was dissolved in a buffer solution and the process was repeated 3–4 times. Removal of monomers was assessed using dynamic light scattering and UV absorption of the supernatant. For the FTIR spectra, 30 µL of the samples were deposited on the silicon crystal of a Bruker Optik Vertex 70 (Ettlingen, Germany) spectrometer. Baseline scans of the buffer solution were taken using 1000 scans at 2 cm^−1^ resolution, and sample spectra were acquired using 700 scans over a range from 3000 to 1000 cm^−1^ wavenumbers. Raw FTIR spectra were normalized to the peak value of their Amide-I band. Difference spectra of RFs and CFs had the normalized monomer spectrum subtracted.

## 3. Results

**Figure 3:**
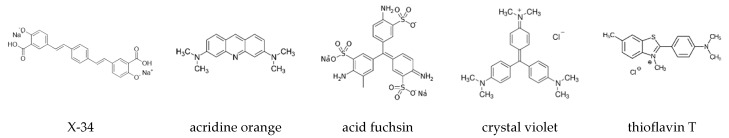
Summary of dye structures. Chemical structure of dyes in this study with indications of oligomer selectivity, together with the reference amyloid indicator dye Thioflavin T.

We monitored a small, select set of dyes for their fluorescence responses to either pure sigmoidal or well-defined biphasic growth conditions. The dyes chosen for this limited screen were either known amyloid dyes, molecular rotors, hydrophobic dyes, or dyes closely associated with our current lead compound—crystal violet. The structure of the dyes we found to display some level of oligomer-selectivity, together with the reference dye thioflavin T, are shown in Figure 3.

**Figure 4:**
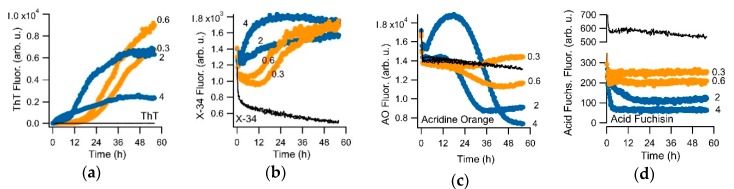
Fluorescence kinetics during sigmoidal and biphasic hewL amyloid assembly for various fluorescent dyes. Kinetics responses of (**a**) thioflavin T, (**b**) the amyloid dye X-34 (λ_ex_ = 370 nm, λ_em_ = 482 nm), (**c**) acridine orange (λ_ex_ = 495 nm, λ_em_ = 532 nm), and (**d**) acid fuchsin (λ_ex_ = 560 nm, λ_em_ = 590 nm) to amyloid growth of hewL at the indicated concentrations (in mg/mL) and under fixed solution conditions (pH 2, 52 °C, 450 mM NaCl). Orange (0.3 & 0.6 mg/mL) versus blue traces (2 & 4 mg/mL) represent sigmoidal versus biphasic growth conditions. Black traces are dye controls recorded from the buffer solution. For overall comparison, ThT traces are displayed on a linear scale, which obscures its weak biphasic response. Dye concentrations were 15 μM. Sharp initial transients resulted from the temperature dependence of dye fluorescence upon heating to 52 °C.

To induce the transition from sigmoidal to biphasic behavior, we either used different salt concentrations while fixing the hewL concentration, or vice versa. Figure 4 compares the sigmoidal versus the biphasic responses of ThT to the responses recorded with the amyloid dye X-34, as well as with acridine orange and acid fuchsin. Each of these dyes showed some sign of selectivity for gO/CFs over RFs. However, either their fluorescence responses were weak (acridine orange) or their selectivity for gO/CFs over RFs were quite modest (acid fuchsin), they displayed excessive bleaching (X-34) or had very high levels of baseline fluorescence (X-34, acridine orange). Screening a rather limited set of dyes did, however, also yield the triarylmethane dye crystal violet (CV) and the closely related methyl violet as promising candidates for oligomer-selective dyes (OSDs). Due to the slightly better signal-to-noise ratio of CV over methyl violet, we focused on CV for our subsequent analysis.

### 3.1. Crystal Violet as Oligomer-Selective Indicator Dye

**Figure 5:**
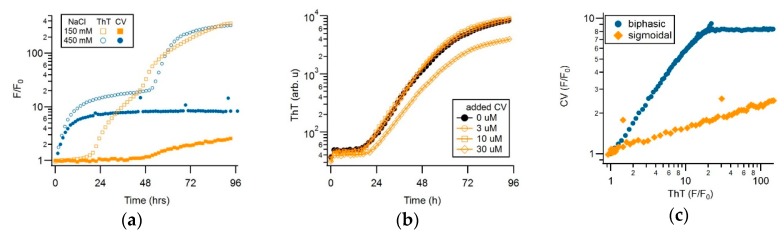
Crystal violet as the oligomer-selective dye (OSD). (**a**) Fractional change of 15 μM ThT (open symbols) versus 5 μM crystal violet (filled symbols) fluorescence during sigmoidal versus biphasic amyloid assembly (350 μM hewL, pH 2, 52 °C) in the presence of either 150 or 400 mM NaCl). (**b**) ThT (15 μM) kinetics of hewL undergoing sigmoidal fibril growth in the presence of increasing concentrations of CV. (**c**) Correlation of fractional CV versus ThT augmentation during biphasic versus sigmoidal growth. During the oligomer-dominated phase of biphasic kinetics, fractional changes of CV and ThT fluorescence are in a lock step and are of comparable magnitude. In contrast, CV becomes essentially unresponsive to the second, fibril-dominated phase. During sigmoidal fibril growth, CV barely increases 1.5-fold, compared to the nearly 100-fold fluorescence increase of ThT. Upon aligning the (noise-limited) CV traces with the upswing in ThT, their responses are strictly linearly correlated in time.

Figure 5a show the superposition of ThT and CV fluorescence responses during sigmoidal (150 mM NaCl) versus biphasic (450 mM NaCl) amyloid growth. To emphasize the dramatic differences in fluorescence responses of the two dyes to sigmoidal versus biphasic amyloid growth, the fractional fluorescence increases F/F_0_ for either dye are displayed on a semi-logarithmic scale. There are several indications that the distinct dye kinetics recorded with CV versus ThT during sigmoidal versus biphasic growth are due to the differential sensitivity of CV to gO/CF versus RF formation. Based on the near-identical 400-fold increase in ThT amplitudes at the end of the 96 h incubation period, the amount of amyloid fibrils present in the 150 versus 450 mM salt solutions are comparable (Figure 5a). In contrast, CV fluorescence in the biphasic regime predominately responded to the initial oligomer-related upswing and with a roughly 10-times larger response than to fibril formation during sigmoidal fibril growth. This difference in CV response persisted irrespective of whether ThT and CV were added to the same sample wells or were measured in separate sample wells, thereby, excluding the contributions from the competitive binding of CV versus ThT to the fibrils. 

The muted CV response to RF formation could potentially arise if CV acts as a select inhibitor of RF formation. The strong ThT response, recorded in the presence of CV under the same condition (see Figure 5a) contradicts that interpretation. To further exclude that CV inhibits RF growth or alters ThT responses, we monitored sigmoidal RF growth for identical hewL/solution conditions, at fixed ThT concentration, but for a series of increasing CV concentrations. As shown in Figure 5b, CV had no discernible effect on the RF kinetics recorded with ThT, up to twice the 5 μM CV concentration used in our typical experiments. At 30 μM of CV, ThT amplitudes were slightly reduced, but with the kinetics of ThT recordings still unchanged. Hence, for the dye concentrations used in our experiments, there was no indication that CV inhibited RF formation. As has been reported for Congo Red and ThT binding to Aβ42 [53], the reduced ThT response observed at 30 μM of CV most likely reflected the competitive binding of ThT and CV to fibrils, instead of the CV-inhibiting RF formation.

**Figure 6:**
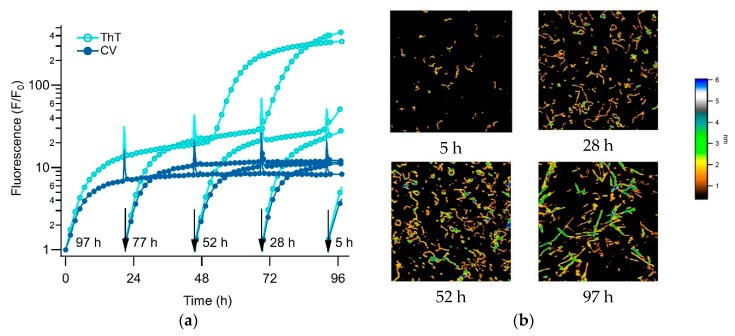
Evolution of amyloid aggregate morphology during biphasic kinetics (**a**) Staggered incubation of 350 μM hewL undergoing biphasic amyloid growth at pH 2, 52 °C, and 450 mM NaCl, recorded simultaneously with 15 μM ThT (●) and 5 μM CV (●). Fresh solutions were added to the 96-well plates at the moments indicated by the arrows. Corresponding total incubation periods are shown next to each arrow. (**b**) AFM images of aliquots imaged from wells incubated for the indicated time periods. While the initial phase of biphasic growth indicates the presence of gOs and increasing numbers of CFs, the late phase shows the simultaneous presence of RFs and CFs, often in direct contact with each other. The false color scale indicates aggregate heights. All images are 3 μm on a side.

Another notable feature of the CV signal during the biphasic growth is its persistence, well into the late-stage RF nucleation and growth period as seen with ThT. We have previously shown that gO/CFs, formed during the initial phase of biphasic kinetics, persist long into the RF nucleation and growth-dominated second phase, including the late-stage plateau in the ThT responses [41]. This reflects the slow rate of gO/CF depolymerization and gradual incorporation into RFs. We reconfirm this prolonged persistence of gO/CFs into the RF growth phase, using a “staggered” incubation protocol—the same biphasic growth solution was added at progressively later times to a temperature-controlled 96-well plate and the accumulated aggregates were collected at a single time-point for imaging. Figure 6a shows the associated series of staggered ThT and CV time traces, together with the correlated AFM images of aggregate morphologies representing different time-points in the assembly reaction. Consistent with our previous observations, the initial phase of biphasic kinetics was dominated by gO/CF formation, while the second phase indicated nucleation and growth of RFs, with residual populations of gO/CFs persisting into the late-stages of our kinetic traces. Hence, the initial rise of CV fluorescence during the biphasic growth correlated with the emergence of gO/CFs. The persistence of CV fluorescence throughout the nucleation and growth of RFs detected by the ThT was supported by the prolonged presence of gO/CFs in the AFM images during that period. 

### 3.2. CV and ThT Report Identical Amyloid Kinetics but with Distinctly Different Sensitivities

**Figure 7:**
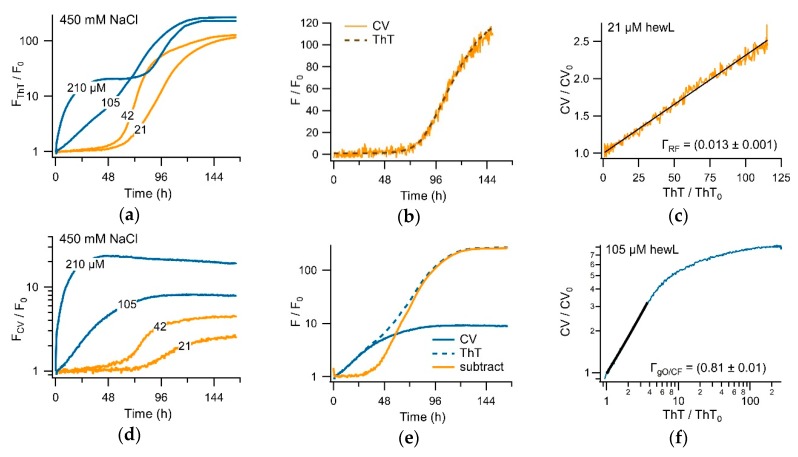
Correlation of CV and ThT kinetics. (**a**,**d**) HewL amyloid formation (pH 2, 450 mM NaCl, T = 52 °C) at concentrations below (orange) and above (blue) the COC, simultaneously monitored with (**a**) ThT (15 μM) and (**d**) CV (5 μM). The traces are the average of the three recordings from separate wells. (**b**,**e**) Superposition of the CV (solid lines) and ThT (dashed lines) responses, after matching the CV data using the Γ-factor determined in (**c**) and (**f**), respectively. Subtracting the matched CV from the ThT trace yields the orange sigmoidal trace in (**e**). (**c**,**f**) Correlation of the fractional changes of CV versus ThT responses during (**c**) sigmoidal versus (**f**) biphasic growth kinetics. The Γ-coefficient indicates the ratio of the CV versus ThT response amplitude in the linear regime of each plot.

Since CV and ThT respond to both gO/CFs and RFs, we evaluated whether both dyes provide identical assembly kinetics but with different sensitivities. For this, we determined the temporal correlation between the CV and the ThT amplitudes under either the sigmoidal or biphasic growth conditions. As shown in Figure 5c, the two dye signals were perfectly correlated in the sigmoidal regime, but with CV showing a much weaker RF response. During the biphasic growth, in contrast, dye responses showed a 1:1 correlation in dye response during the initial gO/CF growth phase, while the two dye signals nearly completely uncoupled during the subsequent RF nucleation and growth phase. To further confirm this preferential selectivity of CV for gO/CFs over RFs, we repeated the experiments by fixing solution conditions and varying the hewL concentrations, instead. Figure 7a,b compared the ThT and CV traces obtained from the same wells. After accounting for their relative difference in sensitivity and a slight shift in initial response time, the fractional CV and ThT increases were perfectly correlated during the sigmoidal RF growth. This perfect correlation in the sigmoidal regime indicated that both dyes detected the same aggregate populations over the entire time course of nucleated RF polymerization. Plotting the fractional ThT versus CV amplitudes for the sigmoidal growth in Figure 7e, further emphasizes that the two dye signals were perfectly linearly correlated over the nearly 7-day incubation period, albeit with very different fluorescence enhancements. The corresponding correlation coefficient of Γ_RF_ = 0.013 indicates that the CV response under these conditions was about 75 times weaker than the ThT response to equivalent concentrations of RFs.

In the biphasic growth regime (blue traces in Figure 7a,b), ThT and CV displayed equivalent kinetics during the oligomer-dominated initial phase, with an overall 20-fold fractional fluorescence enhancement and a linear correlation coefficient of Γ_gO/CF_ = 0.81 ± 0.01. The dramatically lower sensitivity of CV versus ThT to subsequent RF nucleation and growth resulted in the near-complete uncoupling of their respective fluorescence responses in the second, RF-dominated phase. However, the ThT picked up the secondary, fibril-dominated phase (Figure 7a), the low sensitivity of CV to RFs and the concomitant decay of gO/CFs (see Figure 6 above) induced a subtle decay of CV upon the emergence of RFs (Figure 7b). The corresponding amplitude-matched superposition of CV and ThT traces is shown in Figure 7d. The uncoupling of ThT and CV responses in the late phase of biphasic growth is reflected similarly in the sharp break in the correlation of the fractional fluorescence increases for ThT versus CV between the initial and secondary phases of biphasic growth (Figure 7f). 

### 3.3. Separating Amyloid Assembly into its gO/CF and RF Components

The tight temporal correlation of CV and ThT responses to RF formation, combined with their distinctly different sensitivities to gO/CFs over RFs, suggests that simultaneous CV and ThT recordings could permit decomposition of biphasic kinetics into separate gO/CF and RFs growth kinetics. This is important since the temporal evolution of gO/CFs and RFs typically overlap in time and, as we have previously suggested, there are indications that gO/CFs directly interact with and modulate the kinetics of subsequent RF nucleation and growth [41]. To test this, we amplitude-matched the initial oligomer-dominated phase of the CV trace to its corresponding ThT trace (Figure 7d), using their correlation of 0.81 (Figure 7f). As can be seen, the two matched signals coincided perfectly during the initial portion of biphasic growth and then rapidly diverged. The orange curve in Figure 7d is the difference trace resulting from subtracting the matched CV from the ThT signal. Even though this approach ignores the (weak) contributions of the RF formation to the oligomeric CV signal, the difference curve in Figure 7d readily recovers the characteristic sigmoidal kinetics of RF formation—a lag period flat down to the noise level of the fluorescence signal and a rapid autocatalytic upswing nearly reaching the plateau-level of the ThT signal. The blue CV and orange (ThT–CV) difference traces, therefore, represent a near-perfect decomposition of the hewL amyloid kinetics into its separate gO/CF and RF growth components—without any a priori assumption concerning the underlying gO/CF kinetics.

### 3.4. Biphasic Kinetics of Alzheimer Peptides Aβ40 and Aβ42 upon Onset of gO/CF Formation

**Figure 8:**
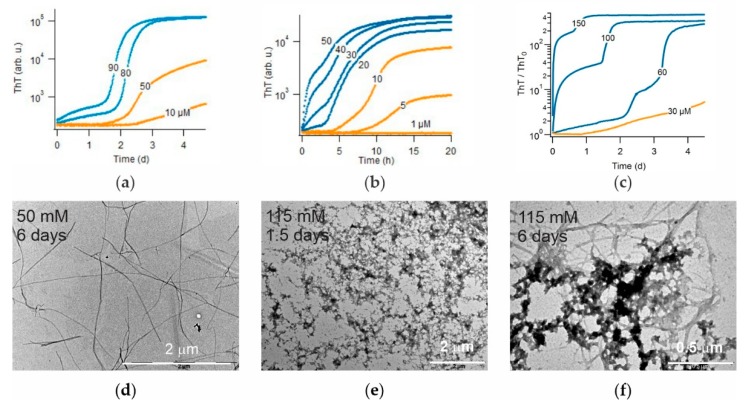
Transition from sigmoidal to biphasic kinetics and associated gO/CF formation for Aβ40 and Aβ42. (**a**) Transition in Aβ40 growth kinetics from pure sigmoidal (orange) to biphasic (blue) kinetics (pH 7.4, no salt). (**b**) Same as (**a**) but for Aβ42. Semilog plot emphasizes weak ThT response during gO/CF phase. (**c**) ThT fractional change during Aβ40 growth in physiological saline. Notice the significant increase in gO/CF amplitude relative to panel (**a**). (**d**–**f**) TEM images of samples of (**a**)—Aβ40 RFs following sigmoidal growth at 50 μM (**d**) versus biphasic growth at 150 μM, with gO/CFs formed within 1.5 days, and (**e**) mixtures of gO/CF and RFs after 6 days (**f**).

There are two intrinsic shortcomings of using hewL to screen for oligomer-selective dyes. First, while hewL is readily available and its assembly kinetics are highly reproducible, it is not a clinically relevant amyloid protein. This shortcoming is mitigated by the observation that the common amyloid stains ThT and Congo Red, as well as some of the newly identified amyloid dyes, often detect amyloids formed by various proteins [38]. The second shortcoming are the non-physiological solution conditions used to observe sigmoidal versus biphasic growth with hewL. As mentioned above, the oligomers formed under those conditions match the features of the toxic oligomers. However, it is well-known that dye responses can vary significantly with solution conditions, such as temperature, pH and ionic strength. However, similar to β2-microglobulin, hewL forms gO/CFs or precipitates near neutral pH—without any discernible RF populations or kinetics to compare to [54]. Hence, we could not extend our kinetic assay to a neutral pH using hewL. We, therefore, explored whether the above kinetic transition could be observed with the clinically relevant Alzheimer Disease peptides Aβ40 and Aβ42, under physiological growth conditions. As shown in Figure 8, both Aβ40 and Aβ42 did display a transition from sigmoidal to biphasic kinetics upon increasing peptide concentration. Using TEM we confirmed that Aβ40 solutions in the sigmoidal regime only yielded fibrillar aggregates (Figure 8d).

Upon the transition to biphasic growth, initial aggregate populations were dominated by highly curvilinear fibrils (CFs) which, even after 6 days, was only partially superseded by RFs (Figure 8e,f). In addition, the initial, oligomeric phase of Aβ40 amyloid growth was further enhanced upon using physiological NaCl concentrations (150 mM). These observations matched the reports of micelle-like oligomer formation in these systems [43,44] and the kinetic measurements in either system [45,55,56]. However, while the gO/CF and RF growth phases of Aβ40 were well-separated by a clear plateau phase (Figure 8a,c), those of Aβ42 showed a strong overlap (Figure 8b). In order to separate the dye responses into their gO/CF versus RF components, a clear temporal separation of the two growth phases is highly desirable. These findings with Aβ40 and Aβ42 are consistent with the switch from oligomer-free sigmoidal to oligomer-dominated biphasic growth that we observed for dimAβ and hewL. Hence, the screening approach presented here can be extended to screen for novel OSDs with Aβ, under physiological solution conditions.

## 4. Discussion

One of the persistent challenges in amyloid research is to unravel the enigmatic role of oligomeric species as either competitors or on-pathway contributors to amyloid fibril assembly, as well as their contributions to the etiology of amyloid diseases in vivo. As pointed out above, part of the challenge is the dependence on antibodies as the exclusive means for detecting and tracking oligomer populations, particularly in in vivo studies. The data presented here suggest that we can identify and validate novel, small oligomer-selective dyes by determining their fluorescence responses to sigmoidal versus biphasic growth. This kinetic approach to dye screening circumvents the need to isolate and stabilize transient and metastable oligomers, in order to screen dyes for their oligomer selectivity. The assay is also robust since both the sigmoidal and biphasic kinetics are highly reproducible for a given choice of solution conditions. Using this approach, we identified the triarylmethane dye crystal violet (CV), as highly selective to lysozyme oligomers over fibrils. We further confirmed that, in the fibril-associated sigmoidal assembly regime, CV yielded kinetics identical to those of ThT—albeit with a significantly lower sensitivity. In contrast, the sensitivity of either dye to the oligomer-dominated initial phase of biphasic growth were nearly identical. While a higher oligomer-sensitivity would be desirable, CV clearly displaced strong selectivity for gO/CFs over RFs in its fluorescence response. This gO/CF selectivity already allowed us to decompose simultaneous CV and ThT recordings of biphasic kinetics into their separate gO/CF and RF components. This should boost efforts in studying the kinetics of oligomer formation versus fibril growth and in the determination of when and how these two processes interact with each other. In addition, this assay could serve as a high throughput screen for monitoring the effects of putative drug candidates on amyloid oligomers versus fibril formation. Perhaps most importantly, though, identifying new small molecules that bind selectivity to oligomers could serve as a starting point in the development of positron-emission tomography (PET) probes, for monitoring the emergence and spread of oligomeric over fibrillar species in neurodegenerative diseases, in vivo. 

## 5. Conclusions

Using the readily available amyloid model hewL, we provided a proof of principle for using sigmoidal versus biphasic kinetics as a screening assay for oligomer-selective dyes. While selective under multiple conditions, we did find that the relative selectivity of CV versus ThT for hewL gO/CFs and RFs did vary with the ionic strength of the growth solution. It is known that the binding of dyes or their fluorescence responses vary with the solution pH and temperature [57,58]. Therefore, for a given amyloid protein, it is important to establish selectivity under physiological growth conditions. To address this concern, we confirmed that the same kinetic transition from sigmoidal to biphasic kinetics is present in Aβ40 and Aβ42 at physiological pH and ionic strength. Hence, we are confident that the screening and validation approach presented here could be extended at least to the Alzheimer’s disease relevant Aβ peptides and are also relevant to other disease-associated amyloid proteins.
